# RRIMS: Radiation Risk In Mammography Screening – a novel model for predicting the lifetime dose and risk of radiation-induced breast cancer from the first screening visit

**DOI:** 10.1259/bjro.20220028

**Published:** 2022-09-29

**Authors:** Sahand Hooshmand, Warren M. Reed, Mo'ayyad E. Suleiman, Patrick C. Brennan

**Affiliations:** 1 Faculty of Medicine and Health, Discipline of Medical Imaging Sciences, The University of Sydney, Susan Wakil Health Building (D18), Sydney, Australia

## Abstract

**Objectives:**

**R**adiation **R**isk **I**n **M**ammography **S**creening (**RRIMS**) builds on the prototype, formerly known as Breast-iRRISC, to develop a model that aims to establish a dose and risk profile for females by calculating their lifetime mean glandular dose (MGD) for each age of screening between 40 and 75 years, using only the information from her first screening visit. This is then used to allocate her to a dose category and estimate the lifetime risk of radiation-induced breast cancer incidence and mortality for a population of females in that category.

**Methods:**

This model training was developed using a large dataset of Hologic images containing a total of 20,232 images from 5,076 visits from 4,154 females. The female’s breast characteristics and exposure parameters were extracted from the images to calculate the female’s MGD throughout a lifetime of screening from just her first screening visit, using modelling of various parameters and their change through time.

**Results:**

This development has ultimately provided a model that uses the female’s first screening visit to calculate the received MGD for all ages of potential screening. This has enabled the allocation of females to either a low-, medium-, or high-dose category, ultimately followed by the lifetime effective risk (LER) estimation for any screening attendance pattern. A female in the low-dose category undergoing biennial screening from 50 to 74 years would expect a risk of radiation-induced breast cancer incidence and mortality of 8.64 and 2.61 cases per 100,000 females, respectively. Similarly, a female in the medium- or high-dose category undergoing the same regimen would expect an incidence and mortality risk of 11.76 and 3.55, and 15.08 and 4.55 cases per 100,000 females, respectively.

**Conclusions:**

This novel approach of establishing a female’s dose profile and lifetime risk from a single visit will further assist females in their informed consent on breast screening attendance and help inform policy-makers when exploring the benefits and drawbacks of various screening patterns and frequencies.

**Advances in knowledge:**

RRIMS is a novel tool that enables the assessment of a female’s lifetime dose and risk profile using only the information from her first screening visit.

## Introduction

Ionising radiation exposure during screening mammography can contribute to a female’s risk of developing breast cancer, adding to their absolute lifetime risk of 1-in-8.^1^ There are many factors that can influence the absorbed dose and subsequent risks involved with screening mammographic procedures. These can be classified into two categories: (a) patient characteristics, such as the female’s age, mammographic breast density (MBD), compressed breast thickness (CBT), and the number of projections required that is dependent upon breast size, and (b) factors related to the mammography unit’s exposure parameters, such as the target/filter (T/F) combination, half value layer (HVL), kVp, and mAs. Although all of these factors play a key role, some contribute more to the calculation and estimation of the dose and risk using the formulations from Dance et al.^2^ and Brenner et al.,^3^ respectively.

The risk of radiation-induced breast cancer from screening mammography is an area that has been previously explored through many different lenses, most notably by Yaffe and Mainprize,^4^ Miglioretti et al.^5^ and M.Ali et al.^6,7^ In 2011, the radiation-induced breast cancer incidence and mortality was calculated for a variety of different screening regimens of annual and biennial screening frequencies, ranging from ages 40 to 74 years by Yaffe and Mainprize. They assumed a set dose of 3.7 milligray (mGy) for both breasts per examination and concluded that for a cohort of 100,000 females who are undergoing annual screening from 40 to 55 years and biennially thereafter to 74 years, it would induce 86 breast cancers and 11 deaths.^4^ In 2016, this approach was expanded upon by Miglioretti et al with additional screening strategies that covered more annual, biennial, and hybrid strategies, classified by breast size. Unlike the previous approach, the examination radiation dose and the number of projections assumed to be required was determined based on the females’s CBT, the threshold of which was set at 7.5 cm.^5^ In 2018, conversion factors proposed by M.Ali et al. enabled the calculation of effective risk from the mean glandular dose (MGD) for various scenarios of annual, biennial, and triennial screening strategies, with a commencement age available from 25 years and assuming a cessation age of 75 years.^6^ This work was an extension of their earlier mathematical model that aimed to calculate the risk involved with screening mammography.^7^

Upon close examination, the above approaches can be improved upon in two key ways: a) using an accurate and variable examination dose throughout the female’s lifetime of screening, reflecting the change in their breast characteristics, and b) expanding on the various screening regimens a female may undergo. Firstly, the above studies use of a set mammographic dose for calculating the risk across all ages of screening, such as the average dose of 3.7 mGy and 2.019 mGy employed by Yaffe and Mainprize^4^ and M.Ali et al.,^6^ respectively. The alternate approach was the randomly sampled dose per view as in Miglioretti et al.^5^ that was based on the Digital Mammographic Imaging Screening Trial (DMIST) distribution, conditional to the female’s CBT and threshold breast size. The limitation of these approaches is that the received dose changes with age and depends on a multitude of factors, not just CBT. This ultimately results in an inaccurate estimation of risk if using a set inflexible dose for all ages of screening or one that is from a random sampling. Secondly, the lack of adaptable screening strategies and frequencies that could be tailored to any female; instead, each study only had a determined set of screening regimens that assume no deviation from that attendance pattern from start to end. For example M.Ali et al.^6^ only provide annual, biennial, and triennial screening assuming a constant cessation age of 75 years, and although Yaffe and Mainprize^4^ and Miglioretti et al.^5^ provide the typical annual and biennial screening strategies they only total to six and eight, respectively.

Given these shortcomings and the limited information on LER, the Breast Individualised Risk of Radiation Induced Screening Cancer (Breast-iRRISC) model prototype was proposed and developed with the aim of assessing and quantifying the risk of radiation-induced breast cancer from screening mammography. This has already been described in a previous publication.^8^ The strength of this approach over the previous literature lies in the accuracy of the risk estimations as it utilises females’ physical breast characteristics and exposure parameters from their presenting Digital Imaging and Communications in Medicine (DICOM) to model their change throughout the ages of screening to assign females to a dose category. It also accounts for the exact history and intended future screening frequency pattern that female is likely to undertake for an individualised estimate of their LER.

The purpose of this **R**adiation **R**isk **I**n **M**ammography **S**creening (**RRIMS**) model was to build on and further train the established Breast-iRRISC prototype model using an independent dataset of images obtained from Lifepool. Lifepool is a platform funded by the Australian National Breast Cancer Foundation (NBCF) that contains for each female the images and information from **multiple** visits to the breast screen program. This resource was used in addition to the Cancer Institute New South Wales (CINSW) database, from which the prototype was built,^8^ and is detailed below. The name has also changed from Breast-iRRISC which emphasised *individualised risk* to RRIMS which looks at *individualised dose* but classifies risk into low, medium, and high categories. This process is described below in the methodology.

## Methods

RRIMS is a novel tool that aims to predict females’ lifetime dose and risk of radiation-induced breast cancer in screening mammography. The prototype of this model (Breast-iRRISC) was initially developed in 2020 using a dataset of mammograms obtained from the CINSW, which consisted of 31,097 images, predominantly 2x mediolateral oblique (MLO) and 2x craniocaudal (CC), from 7,728 females, from a **single** screening visit. The X-ray equipment used to generate these data was from two manufacturers: GE Senographe and Hologic Selenia Dimensions.^8^

This study aims to further train and revise that prototype using additional data by improving upon existing and introducing new predictive figures that are crucial for the accurate calculation of the MGD across all ages of screening. An additional dataset of images obtained from Lifepool, that initially contained a total of 128,469 images with 30,419 examinations (visits) from 7,259 females, ranging from numerous manufacturers: Siemens, Hologic, Fujifilm, Philips/Sectra, and Konica, was analysed. However, due to the limited availability of key information from all manufacturers, in particular the inability to define a clear relationship between kVp and T/F with CBT (Step 3c below), and the accuracy of the female’s estimated MBD (Step 1b below), only the Hologic portion of the Lifepool dataset was used in this study. After filtering to include all the necessary pieces of information from the dataset, which included the female’s age, CBT, exposure factors (kVp and mAs), T/F, manufacturer detector ID, and the associated medical physics quality assurance (QA) reports, the remaining Lifepool dataset contained a total of 9,956 images from 2,496 examinations from 1,125 females, where the data followed these females throughout an average of two to three episodes of screening (some up to six visits), which equates to 4 to 6 years assuming a biennial rate. A portion of this Lifepool data (~1/3^rd^) was set aside for validation purposes, therefore the remaining usable set of images (~2/3^rd^) that was ultimately used in this study included: 6,710 images from 1,681 visits from 758 females.

The addition of the entire Lifepool dataset is a key advantage when combined with the CINSW data as it provides a greater volume of information, particularly when analysing various mammographic factors and how they change through time, all of which were used when creating the figures within this paper necessary for the dose calculations.

The same data limitations mentioned above also applied to the GE data from the CINSW dataset, and as a result only the Hologic portion of the original CINSW dataset was added for use in this paper. The combined dataset used for the training of this model now includes a total of 20,232 images (13,522 CINSW+6,710 Lifepool), consisting of mainly four projections (2x MLO and 2 x CC) for a combined 5,076 visits (3,395 CINSW+1,681 Lifepool) from 4,154 females (3,396 CINSW+758 Lifepool) from a single manufacturer (Hologic Selenia Dimensions).

The following steps were taken to further train the model with the addition of the newly acquired Lifepool data.

## STEP 1: Preparing data

### STEP 1a: Extracting data

The first step was to extract the metadata from the dataset of images, which were compiled into a single comma-separated values (CSV) file format that could then be analysed. The females’ anonymised data were extracted from the DICOM headers using the Yakami DICOM Tools software^9^ and exported to a CSV file using a script in MATLAB (version 9.10, R2021a, Natick, Massachusetts: The MathWorks Inc). These were subsequently imported into Microsoft Excel for analysis.

### STEP 1b: Estimating MBD

LIBRA (Laboratory for Individualized Breast Radiodensity Assessment) software package^10^ was then utilised to estimate the MBD of each female screened, which was then added to the corresponding extracted DICOM data.

Note: The LIBRA software is only trained on Hologic and GE images, meaning it only provides a relatively accurate estimation of the MBD from Hologic images in the Lifepool dataset. This limiting step was the reason a large portion of the Lifepool dataset (particularly the Siemens data comprising ~70%) was unsuitable for this study.

### STEP 1c: Extracting medical physics reports

Mammography units undergo regular evaluation by medical physicists to ensure exposure settings and outputs are within the accepted limits. The generated QA report for each associated mammography unit, supplied by the CINSW and BreastScreen Victoria (BSV) for Lifepool, have key information that was extracted and used to calculate the MGD, the details of which are covered by Suleiman et al.^11^ and Robson et al.^12^

## STEP 2: Calculating MGD for DICOM data

### Step 2a: Identifying necessary variables for MGD calculation

The next step was to calculate the MGD for all available images. The extracted data from Step 1 was utilised to calculate the MGD for each available image. The following equation from Dance et al^2^ demonstrates the necessary variables to calculate the MGD for a 2D mammographic exposure:

$$\begin{array}{c} MGD=Kgcs\left(1\right) \end{array}$$

where K is the incident air kerma, the entrance surface dose in mGy that is calculated using the inverse square law and the machine-specific tube output information from the respective medical physics QA reports that are used throughout the MGD calculations; the *g-*, *c-* and *s-factor* are conversion factors applied to specify the breast characteristics and T/F combinations used: *g-factor* converts *K* to MGD assuming a 50% breast glandularity (MBD) model and depends upon the half value layer (HVL) and CBT, *c-factor* corrects for the female’s actual MBD and depends upon the HVL, CBT, and MBD, *s-factor* corrects for the X-ray spectra used for that exposure and is therefore dependent upon the T/F combination.

### STEP 2b: Calculating K and HVL

The incident air kerma further breaks down to the following equation as detailed by Suleiman in 2018:^13^

$$\begin{array}{c} K=\frac{mAs\times A\times kVp^{n}}{{\left(SID-ISD-CBT\right)}^{2}}\left(2\right) \end{array}$$

where *mAs* and *kVp* are exposure factors, *A* and *n* are constants calculated from the QA data, *SID* is the source (X-ray tube) to image distance, *ISD* is the image to support plate distance, both of which are found in the QA reports, and CBT is compressed breast thickness.

Calculating the MGD in Equation 1 requires collecting all necessary variables in Equation 2 for K in addition to the MBD, CBT, HVL, and T/F for the g-, c-, and s-factors. To summarise: the mAs, kVp, CBT, and T/F are found in the DICOM header and were extracted in Step 1a; the MBD was estimated using LIBRA in Step 1b; the SID and ISD were extracted from, and the A and n constants were calculated using data from the QA reports, the data of which were extracted in Step 1c. The only remaining piece is the HVL required for the g- and c-factors; in which the method of HVL calculation for each image is detailed by Robson et al.^12^

### STEP 2c: Calculating MGD

Once the values in Step 2b were collected for each image, Equation 2 was used to calculate K. The HVL, CBT, MBD, and T/F were used to interpolate the appropriate g-, c-, and s-factors (Equation 1) from their respective lookup tables in Dance et al.^14^ These values were then used to calculate the MGD (Step 3, below) for each image as per Equation 1.

## STEP 3: Predicting MGD for all screening visits

To calculate the MGD for missing data, that is for all other relevant ages of screening between 40 and 75 years, where the female has not had a scan, required key input variables that were taken from the first screening visit. These were extracted in Step 1 from the DICOM, and some were calculated in Step 2, which required the medical physics QA reports. These input variables included: age, MBD, CBT, kVp, mAs, T/F, detector ID, manufacturer, model name, and the calculated K and HVL. The calculation and prediction of the values described in Step 2 for all ages of potential screening from 40 to 75 years, where there are no available data, is key to calculating the lifetime MGD. The relationship and estimation of these values will be explored in Steps 3a to 3e.

### STEP 3a: Estimating future MBD

**Figure 1.**
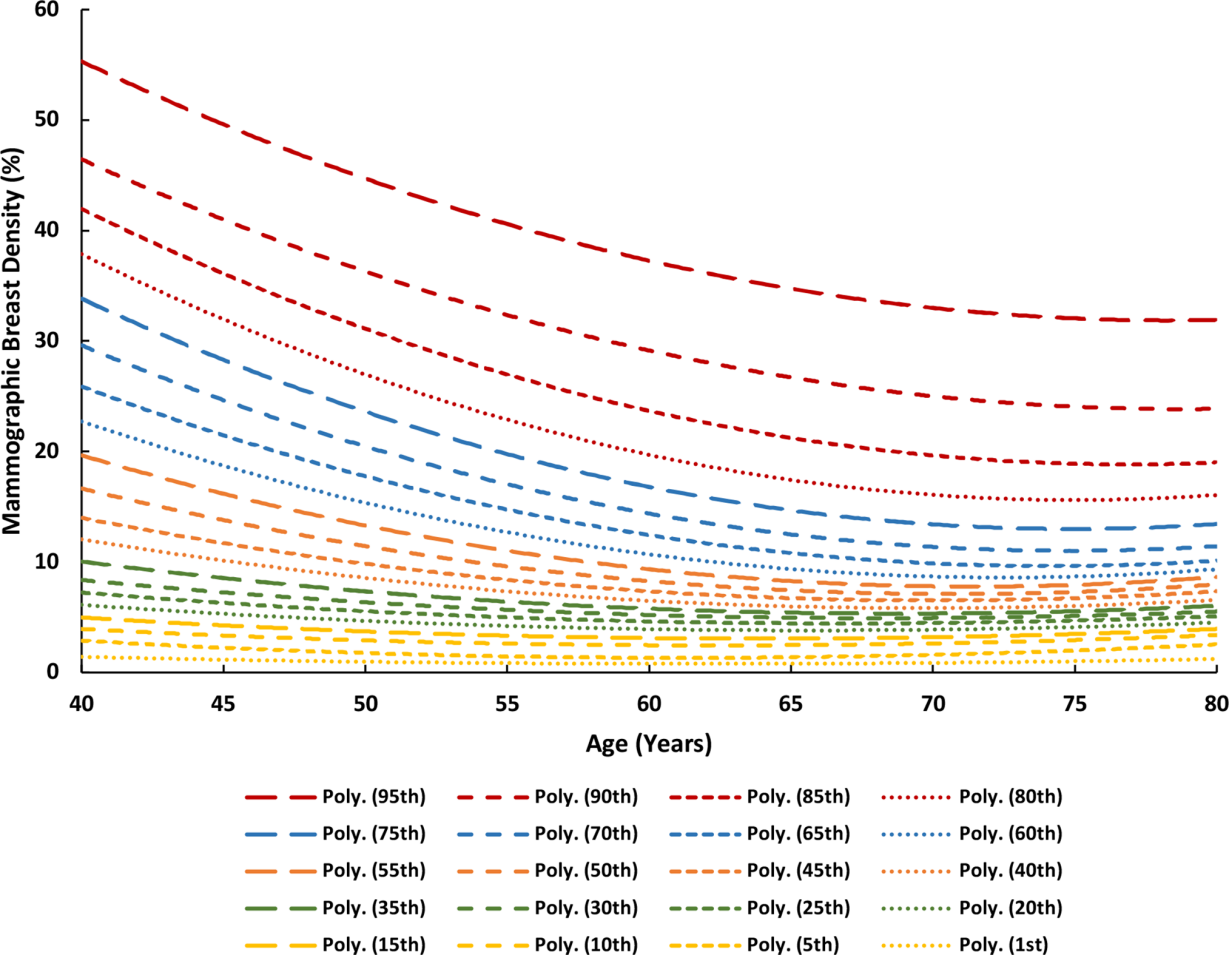
Mammographic breast density (MBD) percentile curves as a function of age in years.

Age was used as a predictor of MBD (explained below); the extracted MBD values were plotted as percentile distribution curves against each age of screening between 40 and 75 years (Figure 1). A second-order polynomial fit is shown for each percentile MBD curve that was derived from Figure 1 with the following form:

$$\begin{array}{c} MBD=aA^{2}+bA+c\left(3\right) \end{array}$$

where *MBD* is the average mammographic breast density as a function of age for the corresponding percentile category of interest, *A* is age in years, and *a*, *b*, and *c* are the fitted coefficients for each of the percentile MBD curves, the values of which are summarised in Table 1.

**Table 1.** Coefficients for each percentile polynomial fit for figures 1, 2 and 4, up to 18 decimal places for the purposes of accurate graph recreation.

		Coefficients			
	Percentiles	a	b	c	d
	**95^th^**	0.0158590893245159	−2.48827364899418	129.468298147669	
**Figure 1**	**90^th^**	0.0150093136786074	−2.36569339840154	117.058387994881	
	**85^th^**	0.0169947953726012	−2.61296241099264	119.2756339392	
	**80^th^**	0.0181604988914373	−2.72430043599688	117.786185105837	
	**75^th^**	0.0170980650793476	−2.56156087824994	108.937944920187	
	**70^th^**	0.0153521524182991	−2.29675134038753	96.9193320105205	
	**65^th^**	0.0139234443090731	−2.06482066303825	86.1942596261178	
	**60^th^**	0.0133474276966086	−1.93586438557909	78.7969203445515	
	**55^th^**	0.0120258656714953	−1.71800473939478	69.1371036864403	
	**50^th^**	0.0101207810347623	−1.43117563671504	57.7055571816715	
	**45^th^**	0.00830370700081973	−1.16267761054262	47.2189394080279	
	**40^th^**	0.00706789465540134	−0.985020366696941	40.1490865369569	
	**35^th^**	0.00567105043709842	−0.780451937085959	32.1851457050436	
	**30^th^**	0.00433908467600172	−0.59183983625958	25.1026783287918	
	**25^th^**	0.00384762323173854	−0.514977208148349	21.6779489863451	
	**20^th^**	0.00350334872002642	−0.460379635671465	18.939769820072	
	**15^th^**	0.00334661384565518	−0.427574160659808	16.7306574543991	
	**10^th^**	0.00297986835672837	−0.371166905786499	14.0175268149771	
	**5^th^**	0.00343005129056988	−0.419996115338768	14.1931566472533	
	**1^st^**	0.00132858467935213	−0.164305179095427	5.8659485827535	
**Figure 2**	**95^th^**	−0.0163082096576135	1.67466808574288	42.1174970236818	
	**90^th^**	−0.0111167901865579	1.0984747527005	52.0927003795982	
	**85^th^**	−0.0112286748985557	1.15848628399392	45.5299695199872	
	**80^th^**	−0.0112498675459536	1.17608166649003	41.8608241446593	
	**75^th^**	−0.0118149009243735	1.25846755343352	36.8016274706577	
	**70^th^**	−0.0127573504204248	1.38474964626977	30.7909264303486	
	**65^th^**	−0.0135713194916254	1.51011757244451	24.4545586007885	
	**60^th^**	−0.0128757799205896	1.44279393890281	24.0804634956656	
	**55^th^**	−0.0135438157984957	1.54067601428636	18.9328224243138	
	**50^th^**	−0.0144265830595951	1.65923874763919	13.5230470040461	
	**45^th^**	−0.0147242150929046	1.71307305853529	9.5036931304673	
	**40^th^**	−0.015604956586238	1.84405820498268	3.37270387015403	
	**35^th^**	−0.0169681424644554	2.0227624615883	−4.29513878067387	
	**30^th^**	−0.0170410701040307	2.06137440551501	−8.25130116621652	
	**25^th^**	−0.0157787830433886	1.91466476660036	−6.20260326740659	
	**20^th^**	−0.0167122724127826	2.05662251531788	−13.7715008757536	
	**15^th^**	−0.0166593687084327	2.05597964570877	−16.829569041281	
	**10^th^**	−0.0133868967107769	1.67625338926533	−9.72284380395601	
	**5^th^**	−0.0117337143854443	1.44402133600941	7.28889567607614	
	**1^st^**	−0.010687280671682	1.24843361091542	−8.58485523551771	
**Figure 4**	**95^th^**	−0.000857981929991203	0.117607691107588	−1.85402559722552	78.2022555926577
	**90^th^**	−0.000592865902141304	0.0764841057193271	−0.355338862532387	56.69267284191
	**85^th^**	−0.000502062808887621	0.0643671787604766	−0.0894005043005408	52.1524594379553
	**80^th^**	−0.000490074790135214	0.0654716660316138	−0.426212402184699	56.8145022678056
	**75^th^**	−0.000489359549370988	0.0678475338520391	−0.775303966044259	62.2361192272739
	**70^th^**	−0.000484375684427441	0.0682825308713444	−0.9253936322067	63.0863675363802
	**65^th^**	−0.000485092288697697	0.0699434860078412	−1.15105112504433	66.0339464074639
	**60^th^**	−0.000475585307884191	0.0695155417310928	−1.24956856606118	67.0777205062206
	**55^th^**	−0.000471103014802327	0.0696613323047411	−1.35030902153644	67.6079394785388
	**50^th^**	−0.000461597593211932	0.0689830527955643	−1.40573625859058	67.6294134547456
	**45^th^**	−0.000466329788241626	0.0705627962797692	−1.55995421317296	68.878678937271
	**40^th^**	−0.000473771586423172	0.0721002418411415	−1.6779633178177	68.9954708286938
	**35^th^**	−0.000479639973981733	0.0742166314915503	−1.8848622068108	71.7688651366056
	**30^th^**	−0.000479175421289918	0.0750857638663224	−2.01487788832269	72.98120227837
	**25^th^**	−0.000487400091693166	0.0771993702161223	−2.17832760193635	73.6611242299634
	**20^th^**	−0.000494545002821492	0.0788870961312435	−2.31763713659349	74.1860736373012
	**15^th^**	−0.000496194521304186	0.0800278149954698	−2.4524220139837	74.7454541201928
	**10^th^**	−0.000501343356137093	0.0817727858706914	−2.62534767391634	75.5292163052138
	**5^th^**	−0.000486317994560698	0.0800148816134606	−2.63124240006265	73.1446354411568
	**1^st^**	−0.000403226727200751	0.0671590191306269	−2.17030695461673	63.3658339605904

**Figure 2.**
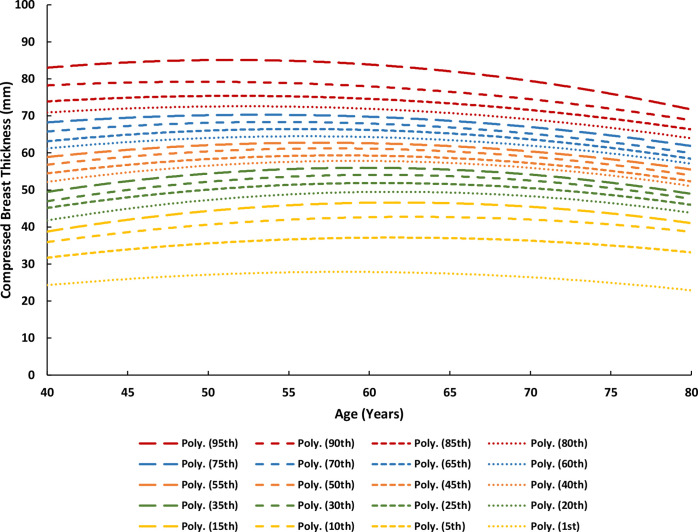
Compressed breast thickness (CBT) percentile curves as a function of age in years.

The female’s MBD from her first screening visit determined which percentile MBD curve to employ. For example, a 55-year-old female presenting with a 10% MBD would prompt the use of the 50^th^ percentile curve (Figure 1), the closest predictor, to estimate that female’s MBD throughout all ages of screening between 40 and 75 years. This replaces the average MBD (*MBD_A_*) and woman-specific MBD (*MBD_WS_*) values and therefore Figures 1 and 2 from the model prototype.^8^

### STEP 3b: Estimating future CBT

Age was also used as a predictor of CBT; the extracted CBT values underwent the same procedure as in Step 2b, with a second-order polynomial fit for each percentile CBT curve derived from Figure 2 with the following form:

$$\begin{array}{c} CBT=aA^{2}+bA+c\left(4\right) \end{array}$$

where *CBT* is the average compressed breast thickness as a function of age for the corresponding percentile category of interest, *A* is age in years, and *a*, *b*, and *c* are the fitted coefficients for each of the percentile MBD curves, the values of which are summarised in Table 1. Analysis of the data suggested that age was a better predictor of CBT than MBD_WS_.

**Figure 3.**
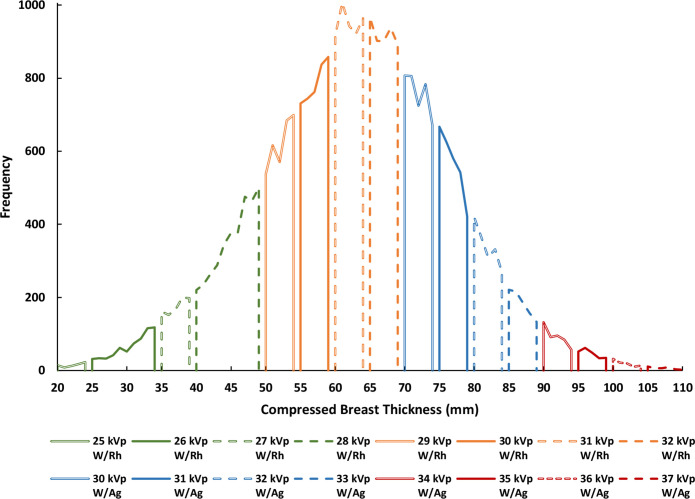
kVp and target/filter (T/F) combination frequency as a function of compressed breast thickness (CBT) for Hologic Selenia Dimensions.

**Figure 4.**
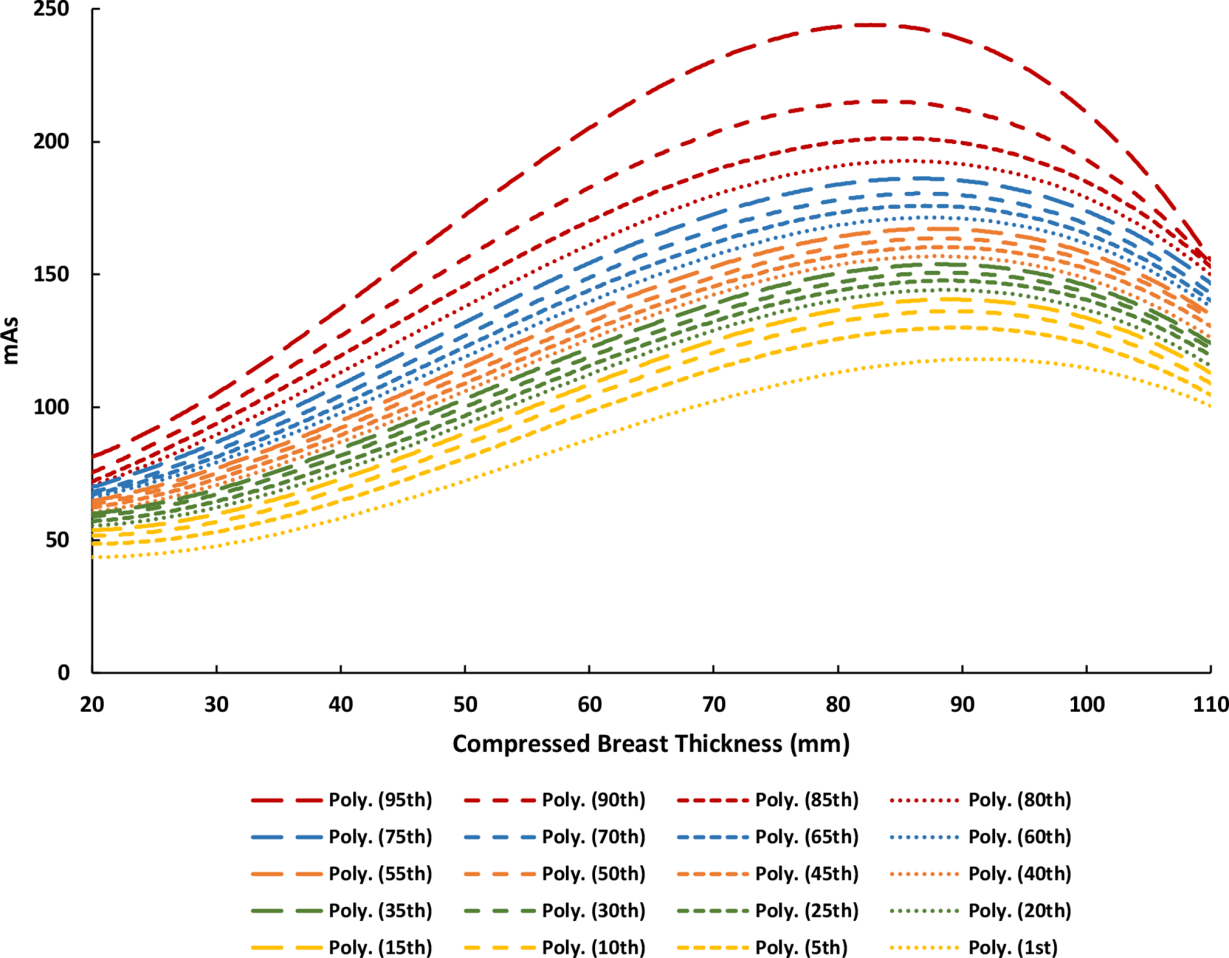
mAs percentile curves as a function of compressed breast thickness (CBT) for Hologic Selenia Dimensions.

The female’s presenting CBT determined which percentile CBT curve to employ. For example, a 60-year-old female presenting with a 70 mm CBT would prompt the use of the 75^th^ percentile curve. This replaces the woman-specific CBT (*CBT_WS_*) and corrected CBT (*CBT_C_*) values and therefore Figures 3 and 4 from the model prototype.^8^

### STEP 3c: Estimating kVp and T/F

CBT was used as a predictor of kVp and T/F. When analysing the frequency distribution of the kVp and T/F against the CBT, the data showed a clear distinction with which values were used for a given CBT (Figure 3). This revealed the kVp and T/F lookup table (Table 2) that a Hologic mammography machine refers to when selecting an exposure, which is based on the female’s CBT. As a result, the data was divided based on the T/F combination used; 20 to 69 mm CBT using a Tungsten/Rhodium (W/Rh) and 70 to 110mm CBT using a Tungsten/Silver (W/Ag). kVp frequency was then plotted as a function of CBT for each T/F combination (Figure 3), showing a clear distinction of which kVp is used for each respective CBT range. kVp and T/F lookup tables were constructed based on these findings (Table 2).

Note: This method of kVp and T/F prediction, which in essence mimics the machine’s kVp and T/F selection, was a new development since the prototype phase. This method of analysis was only compatible with the data from Hologic, Siemens (model: Inspiration), and Fujifilm, of which the Hologic data was used for this paper as it was the common denominator after filtering for the usable data, as described earlier in the methods.

### STEP 3d: Estimating mAs

CBT was also used as a predictor of mAs; the extracted mAs values were plotted as percentile distribution curves as a function of CBT from 20 to 110 mm (Figure 4). A third-order polynomial fit is shown for each percentile mAs curve that was derived from Figure 4 with the following form:

$$\begin{array}{c} mAs=aC^{3}+bC^{2}+cC+d\left(5\right) \end{array}$$

where *mAs* is the average mAs as a function of CBT for the corresponding percentile, *C* is the compressed breast thickness in mm, and *a*, *b*, *c*, and *d* are the fitted coefficients for the respective percentile mAs curves (Table 1).

### STEP 3e: Organising data to calculate lifetime MGD

Once the key values have been calculated for all ages of screening between 40 and 75 years as detailed in Steps 3a to 3d, they are used in Equations 1 and 2 to calculate the MGD for all screening visits, resulting in 36 calculated doses for each female, one for each age of screening between 40 and 75 years. These age-specific dose values were then added together to calculate the total cumulative lifetime dose for that female. Which dose values to add depends on the screening regimen employed, meaning that only those corresponding to the years that female had already attended and those she was likely to attend in the future were included.

The following example will demonstrate this step:

Client A is 56 years old and has only recently considered undertaking routine screening mammography after a conversation with her doctor. She intends to follow BreastScreen Australia’s screening guidelines of biennial screening from 50 to 74 years. Her upcoming mammogram at 56 years will therefore be used as the input and following the calculations in Step 2 she will add 10 different dose values, which correspond to the following ages of screening: 56, 58, 60, 62, 64, 66, 68, 70, 72, and 74 years.

Note: When considering the four standard projections (1x MLO and 1 x CC for both breasts), because the left and right breast are both considered one organ, the dose values are presented as the average of the sum of doses for each breast, however when there was only two projections (1x MLO and 1 x CC for either the left or right breast), then it was assumed the female had undergone a mastectomy, *i.e.,* the two dose values were not averaged and just presented as the sum.

## STEP 4: Dose category

The methods up until now have described how to calculate a female’s received MGD *if* they were to undergo screening every year between 40 and 75. Consequently, this resulted in a list of 36 age-specific MGD values, reflecting the total number of years between 40 and 75 years. These dose values are unique to each individual female as they are completely dependent upon the input variables mentioned in Step 3, which included the female’s age, MBD, CBT, kVp, mAs, T/F, detector ID, and the calculated K and HVL from the X-ray tube manufacturer’s medical physics QA reports.

### STEP 4a: Median MGD per screening round

This step explores how these 36 age- and woman-specific MGD values were used to allocate these females to a dose category. To do this, the key is to use a value that is universal in its calculation method amongst all females in the dataset, despite each individual female having different breast characteristics. The median of all 36 MGD values were taken for each screening visit, and this was referred to as the median MGD per screening round (MGD_MSR_). Given that there were 5,076 examinations that the 4,154 females in the dataset had undergone, this resulted in 5,076 different MGD_MSR_ values. In real life when assessing cumulative lifetime MGD, the range of ages can vary to suit any specific screening regimen, as was covered in Step 3e.

### STEP 4b: Defining dose categories

Depending on the female’s MGD_MSR_, she can now either be allocated to a low-, medium-, or high-dose category, the boundaries of which are defined by the tertile categorisation of all the MGD_MSR_ values across our dataset. The list of 5,076 MGD_MSR_ values, which range from 1.302 mGy to 7.685 mGy, were sorted in ascending order and split at each tertile value; the first 1/3^rd^ constituted the low-dose category, the second 1/3^rd^ constituted the medium-dose category, and the last 1/3^rd^ constituted the high-dose category. This distribution and tertile categorisation of the 5,076 MGD_MSR_ values were used to define the boundary for each dose category; 0 to 3.413 mGy – low, 3.413 to 4.346 mGy – medium, and 4.346 mGy and greater – high.

### STEP 4c: Assigning a dose category

**Figure 5.**
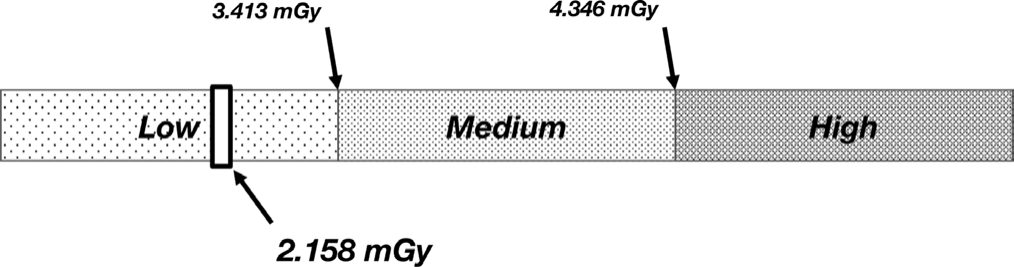
A visual example of the frequency distribution of the median MGD per screening round (MGD where Client A has been assigned to the low-dose category.

The female’s MGD_MSR_ value was then used to assign her to a dose category. The following example will demonstrate this step and serve to summarise the steps taken thus far:

Assume Client A from Step 3e has the following breast characteristics and examination parameters at the age of 56 years, all of which are necessary for the calculations thus far: 26.68% MBD, 40.75 mm CBT, 28 kVp, 88.8 mAs, and a W/Rh T/F combination. *Note that the aforementioned data points are available for each of the four standard projections (2x MLO and 2 x CC), however the values here represent the average of each projection, e.g., the 26.68% MBD is the average MBD across all images taken in the examination that year*. Based on these parameters, she would use the 85^th^ percentile MBD curve (Figure 1), the 10^th^ percentile CBT curve (Figure 2), the 40^th^ percentile mAs curve (Figure 4), a W/Rh T/F, and 28 kVp based on the CBT as per Table 2 (derived from Figure 3). Following the steps thus far using the relevant data, client A would have an MGD_MSR_ of 2.158 mGy, which places her in the low dose category, as illustrated in Figure 5.

**Table 2.** kVP and T/F lookup table based on compressed breast thickness (CBT) range for Hologic Selenia Dimensions.

CBT	kVp	T/F
20–24	25	W/RH
25–34	26	W/RH
35–39	27	W/RH
40–49	28	W/RH
50–54	29	W/RH
55–59	30	W/RH
60–64	31	W/RH
65–69	32	W/RH
70–74	30	W/AG
75–79	31	W/AG
80–84	32	W/AG
85–89	33	W/AG
90–94	34	W/AG
95–99	35	W/AG
100–104	36	W/AG
105–110	37	W/AG

How these dose categories translate to a quantifiable risk of radiation-induced breast cancer incidence and mortality is covered in Step 5.

### STEP 5: Calculating lifetime effective risk for a dose category

Following the allocation of a female to a dose category, as an additional measure, the female can opt to assess the cumulative LER of radiation-induced breast cancer incidence and mortality for a population of females in the assigned low-, medium-, or high-dose category, now considering her personal screening frequency and regimen. The LER is the summation of all the effective risk values, which correspond to the years that the female has attended breast screening. The LER will vary for each case as it ultimately depends on the number of screening visits; this can be influenced by the different screening frequency recommendations of each country, but it is fundamentally based on how often a female chooses to attend. This calculation initially requires the conversion of the MGD values for each relevant age of screening to effective risk, and is done with the following equation from Brenner:^3^

$$\begin{array}{c} R=\sum r_{T}H_{T}\left(6\right) \end{array}$$

where *R* is the effective risk, *r_T_* is the cancer lifetime attributable risk (LAR) for tissue *T*, and *H_T_* is the dose for tissue *T. Note: r_T_ and H_T_ must be in the same dose units, e.g., mGy*.

### STEP 5a: Final median MGD values

To calculate the H_T_ values that would be used in the LER calculations as per Equation 6, the median MGD was calculated for each age of screening between 40 to 75 years for all females in the dose category of interest, *i.e.,* whichever category was assigned in Step 4c. This calculation ultimately resulted in three series of 36 MGD values (one for each age of screening), with each series corresponding to a dose category. There were 1,692 examinations representing each category, which equated to the total of 5,076 screening visits in the entire dataset. These values have been summarised in Table 3.

**Table 3.** The median MGD values per screening round (MGD_MSR_) for the low-, medium-, and high-dose categories for each age of screening between 40 and 75 years.

	Dose Categories		
Age	Low	Medium	High
40	2.445	3.520	4.656
41	2.471	3.551	4.673
42	2.546	3.600	4.709
43	2.559	3.629	4.756
44	2.587	3.653	4.764
45	2.612	3.673	4.779
46	2.667	3.767	4.920
47	2.667	3.776	4.930
48	2.687	3.780	4.928
49	2.761	3.834	4.957
50	2.808	3.860	4.966
51	2.806	3.859	4.979
52	2.852	3.904	5.000
53	2.863	3.914	5.010
54	2.859	3.888	4.955
55	2.863	3.886	4.962
56	2.868	3.901	4.980
57	2.882	3.904	4.981
58	2.886	3.891	4.953
59	2.900	3.929	5.029
60	2.902	3.928	5.036
61	2.900	3.930	5.032
62	2.905	3.942	5.058
63	2.906	3.938	5.056
64	2.903	3.936	5.066
65	2.912	3.953	5.080
66	2.914	3.960	5.084
67	2.910	3.907	5.004
68	2.879	3.906	5.069
69	2.870	3.893	5.068
70	2.868	3.868	5.024
71	2.865	3.842	4.952
72	2.812	3.805	4.902
73	2.808	3.783	4.880
74	2.811	3.817	4.987
75	2.789	3.777	4.886
**Median**	**2.861**	**3.877**	**4.972**

### STEP 5b: Calculating LAR

**Figure 6.**
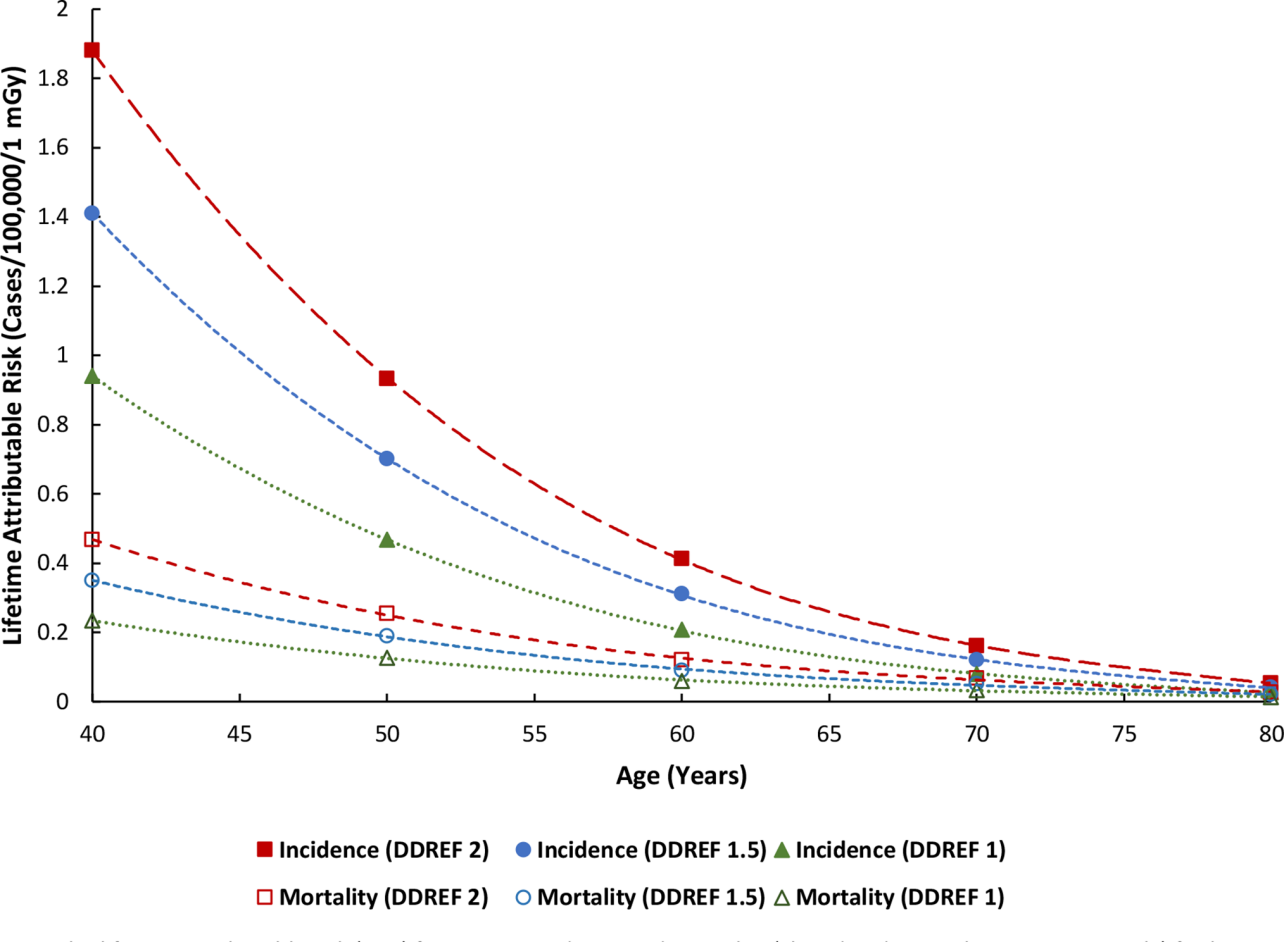
The lifetime attributable risk (LAR) for cancer incidence and mortality (closed and open shapes, respectively) for breast tissue per 100,000 females exposed to a single dose of 1 mGy for a DDREF of 1 (triangle), 1.5 (circle) and 2 (square). Original source: Health Risks from Exposure to Low Levels of Ionising Radiation: BEIR VII.

The LAR is a population-based estimate of radiation risk which uses conversion factors to translate dose (in mGy) to risk. These LAR conversion factors are available for each organ site for males and females, for cancer incidence and mortality, within the Health Risks from Exposure to Low Levels of Ionising Radiation: BEIR VII Phase two report.^15^ However the focus for the current paper was on the LAR for breast tissue, where the original values, along with the interpolated age and the dose and dose rate effectiveness factor (DDREF)-specific values are presented in Table 4 and visualised in Figure 6. A third-order polynomial fit is shown for each LAR curve that was derived from Figure 6, to interpolate the r_T_ values for all ages of screening, for cancer incidence (LAR_I_) and mortality (LAR_M_), for a DDREF of 1, 1.5, and 2, with the following form:

$$\begin{array}{c} LAR=aA^{3}+bA^{2}+cA+d\left(7\right) \end{array}$$

**Table 4.** Interpolated lifetime attributable risk (LAR) values for each dose and dose rate effectiveness factor (DDREF). Original source: Health Risks from Exposure to Low Levels of Ionising Radiation: BEIR VII Phase two report

		Cancer Incidence Per 100,000 Per 0.1 Gy (Original)	Cancer Incidence Per 100,000 Per 1 mGy (Interpolated)			Cancer Mortality Per 100,000 Per 0.1 Gy (Original)	Cancer Mortality Per 100,000 Per 1 mGy (Interpolated)		
	DDREF	1.5	1	1.5	2	1.5	1	1.5	2
**Age at exposure**	40	141	2.1143571429	1.4095714286	1.0571785714	35	0.5260714286	0.3507142857	0.2630357143
	41		1.9825237500	1.3216825000	0.9912618750		0.4956937500	0.3304625000	0.2478468750
	42		1.8568114286	1.2378742857	0.9284057143		0.4667142857	0.3111428571	0.2333571429
	43		1.7370626786	1.1580417857	0.8685313393		0.4390955357	0.2927303571	0.2195477679
	44		1.6231200000	1.0820800000	0.8115600000		0.4128000000	0.2752000000	0.2064000000
	45		1.5148258929	1.0098839286	0.7574129464		0.3877901786	0.2585267857	0.1938950893
	46		1.4120228571	0.9413485714	0.7060114286		0.3640285714	0.2426857143	0.1820142857
	47		1.3145533929	0.8763689286	0.6572766964		0.3414776786	0.2276517857	0.1707388393
	48		1.2222600000	0.8148400000	0.6111300000		0.3201000000	0.2134000000	0.1600500000
	49		1.1349851786	0.7566567857	0.5674925893		0.2998580357	0.1999053571	0.1499290179
	50	70	1.0525714286	0.7017142857	0.5262857143	19	0.2807142857	0.1871428571	0.1403571429
	51		0.9748612500	0.6499075000	0.4874306250		0.2626312500	0.1750875000	0.1313156250
	52		0.9016971429	0.6011314286	0.4508485714		0.2455714286	0.1637142857	0.1227857143
	53		0.8329216071	0.5552810714	0.4164608036		0.2294973214	0.1529982143	0.1147486607
	54		0.7683771429	0.5122514286	0.3841885714		0.2143714286	0.1429142857	0.1071857143
	55		0.7079062500	0.4719375000	0.3539531250		0.2001562500	0.1334375000	0.1000781250
	56		0.6513514286	0.4342342857	0.3256757143		0.1868142857	0.1245428571	0.0934071429
	57		0.5985551786	0.3990367857	0.2992775893		0.1743080357	0.1162053571	0.0871540179
	58		0.5493600000	0.3662400000	0.2746800000		0.1626000000	0.1084000000	0.0813000000
	59		0.5036083929	0.3357389286	0.2518041964		0.1516526786	0.1011017857	0.0758263393
	60	31	0.4611428571	0.3074285714	0.2305714286	9	0.1414285714	0.0942857143	0.0707142857
	61		0.4218058929	0.2812039286	0.2109029464		0.1318901786	0.0879267857	0.0659450893
	62		0.3854400000	0.2569600000	0.1927200000		0.1230000000	0.0820000000	0.0615000000
	63		0.3518876786	0.2345917857	0.1759438393		0.1147205357	0.0764803571	0.0573602679
	64		0.3209914286	0.2139942857	0.1604957143		0.1070142857	0.0713428571	0.0535071429
	65		0.2925937500	0.1950625000	0.1462968750		0.0998437500	0.0665625000	0.0499218750
	66		0.2665371429	0.1776914286	0.1332685714		0.0931714286	0.0621142857	0.0465857143
	67		0.2426641071	0.1617760714	0.1213320536		0.0869598214	0.0579732143	0.0434799107
	68		0.2208171429	0.1472114286	0.1104085714		0.0811714286	0.0541142857	0.0405857143
	69		0.2008387500	0.1338925000	0.1004193750		0.0757687500	0.0505125000	0.0378843750
	70	12	0.1825714286	0.1217142857	0.0912857143	5	0.0707142857	0.0471428571	0.0353571429
	71		0.1658576786	0.1105717857	0.0829288393		0.0659705357	0.0439803571	0.0329852679
	72		0.1505400000	0.1003600000	0.0752700000		0.0615000000	0.0410000000	0.0307500000
	73		0.1364608929	0.0909739286	0.0682304464		0.0572651786	0.0381767857	0.0286325893
	74		0.1234628571	0.0823085714	0.0617314286		0.0532285714	0.0354857143	0.0266142857
	75		0.1113883929	0.0742589286	0.0556941964		0.0493526786	0.0329017857	0.0246763393
	76		0.1000800000	0.0667200000	0.0500400000		0.0456000000	0.0304000000	0.0228000000
	77		0.0893801786	0.0595867857	0.0446900893		0.0419330357	0.0279553571	0.0209665179
	78		0.0791314286	0.0527542857	0.0395657143		0.0383142857	0.0255428571	0.0191571429
	79		0.0691762500	0.0461175000	0.0345881250		0.0347062500	0.0231375000	0.0173531250
	80	4	0.0593571429	0.0395714286	0.0296785714	2	0.0310714286	0.0207142857	0.0155357143

where *LAR* is the lifetime attributable risk in cases per 100,000 females for a single exposure of 1 mGy, *A* is age in years, and *a*, *b*, *c*, and *d* are the fitted coefficients for each LAR curve, the values of which are summarised in Table 5.

**Table 5.** Coefficients for each lifetime attributable risk (LAR) polynomial fit for Figure 6, up to 20 decimal places for the purposes of accurate graph recreation

		Coefficients			
	DDREF	a	b	c	d
**Incidence (LAR_I_**)	1	−0.0000262499999999986	0.00628928571428544	−0.512089285714269	14.2150714285711
	1.5	−0.0000174999999999991	0.00419285714285698	−0.341392857142847	9.47671428571409
	2	−0.0000131249999999993	0.00314464285714272	−0.256044642857134	7.10753571428554
**Mortality (LAR_M_**)	1	−0.00000624999999999958	0.00146785714285707	−0.118517857142853	3.31821428571419
	1.5	−0.00000416666666666648	0.000978571428571391	−0.0790119047619024	2.21214285714281
	2	−0.00000312499999999979	0.000733928571428534	−0.0592589285714263	1.6591071428571

### STEP 5c: Calculating LER

The dose category specific MGD values (for each age) from Step 5a (Table 3) and the corresponding age-associated LAR values from Step 5b (Table 4) were used to calculate the effective risk as per Equation 6 for each age of screening between 40 and 75 years. The effective risk values can then be added together, corresponding to the female’s screening pattern and frequency, to estimate her total risk from screening mammography. The summation of all the relevant effective risk values is referred to as the LER for that female’s allocated dose category.

*Note: This is not calculating the lifetime risk of that particular female, but the average risk of all females in that category. This is because the LAR is a population-based parameter and is therefore not suitable for an individual assessment of dose to risk, particularly because there are factors other than radiation that can contribute to a female’s risk of breast cancer throughout her lifetime.*

The following example will demonstrate this step:

Client A was allocated to a low-dose category in Step 4, and she now wants to investigate her LER of radiation-induced breast cancer incidence and mortality given her chosen screening pattern of attendance. First, the median MGD would be retrieved for all females in that low-dose category for the following ages of screening: 56, 58, 60, 62, 64, 66, 68, 70, 72, and 74 years, which correspond to her specific screening attendance (see Step 3e). In this example, the MGD values would be: 2.868, 2.886, 2.902, 2.905, 2.903, 2.914, 2.879, 2.868, 2.812, and 2.811 mGy, respectively (Table 3). Second, the LAR values for cancer incidence and mortality were calculated with Equation 7, using the coefficients in Table 5, for a DDREF of 1, 1.5, and 2. Lastly, the median MGD and age-associated LAR values for both cancer incidence (LAR_I_) and mortality (LAR_M_) were multiplied together for each age of screening and added together as per Equation 6 to calculate her LER.

We have presented the LAR_I_ and LAR_M_ values and the derived equation coefficients in Tables 4 and 5, respectively, for the DDREFs of 1, 1.5, and 2, given the current lack of universal agreement about the optimum value.^16^ This is a simple weighting factor ratio that is applied to the original LAR data from the BEIR VII phase two report, and in this step the DDREF specific LAR_I_ and LAR_M_ values were selected from Table 4.

### STEP 5 summary

The following dot points summarise the risk calculations:

- Each female was allocated to either a low-, medium-, or high-dose category based on her median MGD per screening round (MGD_MSR_) value, across potentially 36 screening rounds (maximum number of screenings).
- The corresponding DDREF specific LAR values are then applied to each of the 36 MGD values to calculate the effective risk for both incidence and mortality for the population of females in the low-, medium-, and high-dose categories.
- The risks of radiation-induced breast cancer incidence and mortality are then simply the sum of the risks across the visits relevant to the specific screening regimen that female adheres to.

## Results

The LER of radiation-induced breast cancer incidence and mortality, in cases per 100,000 females exposed, is summarised in Table 6. These results have been calculated for the low-, medium-, and high-dose categories, as detailed in Step 5 of the methods, for a DDREF of 1, 1.5, and 2, based on the screening regimens of the national breast screening programs of Australia, the UK, and the USA.

**Table 6.** The lifetime effective risk (LER) of radiation-induced breast cancer incidence and mortality in cases per 100,000 females, for the low-, medium-, and high-dose categories, presented for the attendance at various national breast screening programs in Australia, the UK, and the USA

Dose Category	Screening Regimen	Screening Frequency	No. of Visits	DDREF	Cancer Incidence (cases/100,000)	Cancer Mortality (cases/100,000)
Low	Australia	Annually: 40 to 49 years Biennially: 50 to 74 years	23	1	58.36	15.69
				1.5	38.91	10.46
				2	29.18	7.84
		Biennially: 50 to 74 years	13	1	17.27	5.22
				1.5	11.52	3.48
				2	8.64	2.61
	United Kingdom	Triennially: 50 to 74 years	7	1	11.28	3.30
				1.5	7.52	2.20
				2	5.64	1.65
	United States	Annually: 45 to 54 years Biennially: ≥55 (to 75 years)	21	1	41.25	11.56
				1.5	27.50	7.71
				2	20.63	5.78
Medium	Australia	Annually: 40 to 49 years Biennially: 50 to 74 years	23	1	81.73	21.93
				1.5	54.49	14.62
				2	40.87	10.97
		Biennially: 50 to 74 years	13	1	23.51	7.10
				1.5	15.67	4.73
				2	11.76	3.55
	United Kingdom	Triennially: 50 to 74 years	7	1	15.38	4.50
				1.5	10.25	3.00
				2	7.69	2.25
	United States	Annually: 45 to 54 years Biennially: ≥55 (to 75 years)	21	1	56.98	15.94
				1.5	37.99	10.63
				2	28.49	7.97
High	Australia	Annually: 40 to 49 years Biennially: 50 to 74 years	23	1	106.31	28.51
				1.5	70.87	19.01
				2	53.16	14.26
		Biennially: 50 to 74 years	13	1	30.15	9.10
				1.5	20.10	6.07
				2	15.08	4.55
	United Kingdom	Triennially: 50 to 74 years	7	1	19.73	5.78
				1.5	13.15	3.85
				2	9.87	2.89
	United States	Annually: 45 to 54 years Biennially: ≥55 (to 75 years)	21	1	73.56	20.57
				1.5	49.04	13.72
				2	37.78	10.29

A female in the low-dose category undergoing biennial screening from 50 to 74 years would expect a risk of radiation-induced breast cancer incidence and mortality of 8.64 and 2.61 cases per 100,000 females, respectively. Similarly, a female in the medium- or high-dose category undergoing the same regimen would expect an incidence and mortality risk of 11.76 and 3.55, and 15.08 and 4.55 cases per 100,000 females, respectively (Table 6). These values correspond to the calculations made with a DDREF of 2, that is promoted by the ICRP.^17^ This screening regimen, which constitutes 13 total examinations, equates to an increased risk of about 0.009%, 0.012%, and 0.015% for cancer incidence, and 0.003%, 0.004%, and 0.005% for cancer mortality, corresponding to the low-, medium-, and high-dose categories, respectively.

Table 6 only provides the risk values for the most common screening regimens that females in these countries are expected to follow, however any custom screening frequency and pattern can also be applied using the values in Table 7 for a more nuanced approach to the female’s LER assessment within each dose category.

**Table 7.** The risk values in cases per 100,000 females, for each dose category, for a dose and dose rate effectiveness factor (DDREF) or 1, 1.5, and 2

	Cancer Incidence									Cancer Mortality								
**Dose Category**	**Low**			**Medium**			**High**			**Low**			**Medium**			**High**		
**DDREF**	**1**	**1.5**	**2**	**1**	**1.5**	**2**	**1**	**1.5**	**2**	**1**	**1.5**	**2**	**1**	**1.5**	**2**	**1**	**1.5**	**2**
**Age**	**Cases per 100,000** **females**																	
**40**	5.170	3.447	2.585	7.441	4.961	3.721	9.845	6.563	4.922	1.286	0.858	0.643	1.852	1.234	0.926	2.449	1.633	1.225
**41**	4.900	3.266	2.450	7.041	4.694	3.520	9.264	6.176	4.632	1.225	0.817	0.613	1.760	1.174	0.880	2.316	1.544	1.158
**42**	4.727	3.151	2.364	6.684	4.456	3.342	8.743	5.829	4.371	1.188	0.792	0.594	1.680	1.120	0.840	2.198	1.465	1.099
**43**	4.444	2.963	2.222	6.303	4.202	3.152	8.261	5.507	4.131	1.123	0.749	0.562	1.593	1.062	0.797	2.088	1.392	1.044
**44**	4.200	2.800	2.100	5.929	3.952	2.964	7.733	5.155	3.867	1.068	0.712	0.534	1.508	1.005	0.754	1.967	1.311	0.983
**45**	3.956	2.637	1.978	5.564	3.710	2.782	7.240	4.827	3.620	1.013	0.675	0.506	1.424	0.950	0.712	1.853	1.236	0.927
**46**	3.765	2.510	1.883	5.319	3.546	2.660	6.946	4.631	3.473	0.971	0.647	0.485	1.371	0.914	0.686	1.791	1.194	0.895
**47**	3.507	2.338	1.753	4.964	3.310	2.482	6.481	4.320	3.240	0.911	0.607	0.455	1.290	0.860	0.645	1.683	1.122	0.842
**48**	3.284	2.189	1.642	4.621	3.080	2.310	6.023	4.016	3.012	0.860	0.573	0.430	1.210	0.807	0.605	1.577	1.052	0.789
**49**	3.134	2.089	1.567	4.352	2.901	2.176	5.626	3.751	2.813	0.828	0.552	0.414	1.150	0.767	0.575	1.486	0.991	0.743
**50**	2.956	1.970	1.478	4.063	2.709	2.032	5.227	3.485	2.613	0.788	0.525	0.394	1.084	0.722	0.542	1.394	0.929	0.697
**51**	2.735	1.823	1.368	3.762	2.508	1.881	4.854	3.236	2.427	0.737	0.491	0.368	1.013	0.676	0.507	1.308	0.872	0.654
**52**	2.572	1.715	1.286	3.520	2.347	1.760	4.508	3.006	2.254	0.700	0.467	0.350	0.959	0.639	0.479	1.228	0.819	0.614
**53**	2.385	1.590	1.192	3.260	2.173	1.630	4.173	2.782	2.086	0.657	0.438	0.329	0.898	0.599	0.449	1.150	0.766	0.575
**54**	2.197	1.465	1.098	2.987	1.991	1.494	3.807	2.538	1.904	0.613	0.409	0.306	0.833	0.556	0.417	1.062	0.708	0.531
**55**	2.027	1.351	1.013	2.751	1.834	1.376	3.513	2.342	1.756	0.573	0.382	0.287	0.778	0.519	0.389	0.993	0.662	0.497
**56**	1.868	1.245	0.934	2.541	1.694	1.271	3.243	2.162	1.622	0.536	0.357	0.268	0.729	0.486	0.364	0.930	0.620	0.465
**57**	1.725	1.150	0.862	2.337	1.558	1.168	2.982	1.988	1.491	0.502	0.335	0.251	0.680	0.454	0.340	0.868	0.579	0.434
**58**	1.586	1.057	0.793	2.138	1.425	1.069	2.721	1.814	1.360	0.469	0.313	0.235	0.633	0.422	0.316	0.805	0.537	0.403
**59**	1.461	0.974	0.730	1.979	1.319	0.989	2.533	1.688	1.266	0.440	0.293	0.220	0.596	0.397	0.298	0.763	0.508	0.381
**60**	1.338	0.892	0.669	1.812	1.208	0.906	2.322	1.548	1.161	0.410	0.274	0.205	0.556	0.370	0.278	0.712	0.475	0.356
**61**	1.223	0.816	0.612	1.658	1.105	0.829	2.123	1.415	1.061	0.383	0.255	0.191	0.518	0.346	0.259	0.664	0.442	0.332
**62**	1.120	0.746	0.560	1.519	1.013	0.760	1.950	1.300	0.975	0.357	0.238	0.179	0.485	0.323	0.242	0.622	0.415	0.311
**63**	1.023	0.682	0.511	1.386	0.924	0.693	1.779	1.186	0.890	0.333	0.222	0.167	0.452	0.301	0.226	0.580	0.387	0.290
**64**	0.932	0.621	0.466	1.264	0.842	0.632	1.626	1.084	0.813	0.311	0.207	0.155	0.421	0.281	0.211	0.542	0.361	0.271
**65**	0.852	0.568	0.426	1.157	0.771	0.578	1.486	0.991	0.743	0.291	0.194	0.145	0.395	0.263	0.197	0.507	0.338	0.254
**66**	0.777	0.518	0.388	1.055	0.704	0.528	1.355	0.903	0.678	0.271	0.181	0.136	0.369	0.246	0.184	0.474	0.316	0.237
**67**	0.706	0.471	0.353	0.948	0.632	0.474	1.214	0.810	0.607	0.253	0.169	0.127	0.340	0.226	0.170	0.435	0.290	0.218
**68**	0.636	0.424	0.318	0.863	0.575	0.431	1.119	0.746	0.560	0.234	0.156	0.117	0.317	0.211	0.159	0.411	0.274	0.206
**69**	0.576	0.384	0.288	0.782	0.521	0.391	1.018	0.679	0.509	0.217	0.145	0.109	0.295	0.197	0.147	0.384	0.256	0.192
**70**	0.524	0.349	0.262	0.706	0.471	0.353	0.917	0.612	0.459	0.203	0.135	0.101	0.274	0.182	0.137	0.355	0.237	0.178
**71**	0.475	0.317	0.238	0.637	0.425	0.319	0.821	0.548	0.411	0.189	0.126	0.095	0.253	0.169	0.127	0.327	0.218	0.163
**72**	0.423	0.282	0.212	0.573	0.382	0.286	0.738	0.492	0.369	0.173	0.115	0.086	0.234	0.156	0.117	0.301	0.201	0.151
**73**	0.383	0.255	0.192	0.516	0.344	0.258	0.666	0.444	0.333	0.161	0.107	0.080	0.217	0.144	0.108	0.279	0.186	0.140
**74**	0.347	0.231	0.174	0.471	0.314	0.236	0.616	0.410	0.308	0.150	0.100	0.075	0.203	0.135	0.102	0.265	0.177	0.133
**75**	0.311	0.207	0.155	0.421	0.280	0.210	0.544	0.363	0.272	0.138	0.092	0.069	0.186	0.124	0.093	0.241	0.161	0.121

## Discussion

### Summary and main findings

Quantifying the amount, accumulation, and putting into perspective the associated risks of the ionising-radiation used within screening mammography, alongside the benefits of that procedure, is paramount to a female’s understanding and informed consent discussion they have with their families and medical advisors. This model serves to further inform females to better understand the benefits and risks associated with a procedure designed to save lives using ionising radiation.

The output of RRIMS is twofold: first, it works to assign a female to either a low, medium, or high dose category, which serves to provide a quick and easily understandable assessment of their dose profile, and second, it calculates the LER of radiation-induced breast cancer incidence and mortality for the population of females in their particularly assigned dose category, which is tailored to the timing of the individual female’s screening regimen. The strength of this model is that this can be achieved using only the information from the female’s first screening visit, thus generating risk data at the earliest point of a screening journey. This paper documents the developments made since the initial prototype (Breast-iRRISC), as it uses additional data to create a more accurate model. This has enabled a new and improved method for predicting the mAs (Figure 4), kVp, and associated T/F usage (Table 2 and Figure 3) with the female’s CBT, in addition to facilitating a complete revision of the original MBD (Figure 1) and CBT (Figure 2) percentile curves.

The results from this trained model are summarised in Table 6. Overall, they indicate that breast screening has a very small level of increased risk of radiation-induced breast cancer incidence and mortality when comparing either of the low-, medium-, or high-dose categories. Taking the example of a female in the medium-dose category, undergoing BreastScreen Australia’s recommendation of biennial screening from 50 to 74 years (*i.e.,* 13 examinations), her calculated increased risk is about 11.76 and 3.55 cases per 100,000 females for breast cancer incidence and mortality, which equates to an increased risk of 0.012 and 0.004%, respectively. Even when considering the optional additional annual screening from 40 to 49 years (*i.e.,* now 23 examinations) that BreastScreen Australia offers females free of charge, the corresponding risk is 40.87 (0.041%) and 10.97 (0.011%) cases per 100,000 females, for breast cancer incidence and mortality, respectively.

To put this into perspective, according to the latest Surveillance Epidemiology and End Results (SEER) data from 2017, the average female in the U.S. has a 0.07%, 0.49%, 1.55%, 2.40%, 3.54%, and 4.09% risk of being diagnosed with breast cancer within the next 10 years, at a starting age of 20, 30, 40, 50, 60, and 70 years, respectively.^18^ For example a female who is currently 60 years old would be expected to have a 3.54% (1-in-28) chance of getting breast cancer over the next decade, assuming she is alive and cancer-free at her current age. The SEER data suggest that the average female has an overall 1-in-8 (12.14%) chance of being diagnosed with breast cancer in her lifetime. When comparing the 0.012% increased risk of breast cancer incidence resulting from biennial screening from 50 to 74 years (*i.e.,* 13 examinations in total), the female’s inherent baseline lifetime risk of 12.14% is over 1000 times higher.

### Importance and relevance

The contributions from this piece of research have the potential to be highly valuable to the reader and community of females particularly within the age bracket eligible for regular screening mammography. It provides information that can empower females with the knowledge and perspective required to make an informed decision, under the guidance of their medical advisors, about their choice concerning what regimen to adopt at breast cancer screening programs. On a governance level, it can also inform policy on screening age range recommendations by analysing the risk profile of the various start and end combinations of a female’s age that may undergo screening mammography. This is all made possible by providing this accurate method of dose calculation that is primarily used to allocate females to a dose category, but that also can be used to calculate the lifetime risk of radiation-induced breast cancer incidence and mortality for a population of females in each category.

Although this novel individualised approach to dose and risk calculation is unique, it is still possible to compare the results from this paper with that from previous research, once the screening parameters are aligned. For example, in 2011, Yaffe and Mainprize^4^ claimed that per 100,000 females exposed to a constant dose of 3.7 mGy for annual screening from 40 to 55 years, and then biennially until 74 years, the increased lifetime risk would be 86 induced cancers and 11 deaths. Comparing this to our model, when factoring in the same screening regimen and parameters, there would be 90 induced cancers and 25 deaths per 100,000 females screened. In 2016, Miglioretti et al.^5^ provided the lifetime risk of radiation-induced breast cancer, this time with the radiation dose per view randomly sampled based on the DMIST distribution, which was dependent upon the female’s CBT. They found that on average screening annually from 40 to 74 years would result in 125 breast cancer cases and 16 deaths per 100,000 females exposed, which when comparing this lifetime risk from our model, there would be 103 cancer cases and 28 deaths per 100,000 females. In 2018, M.Ali et al.^6^ provided a list of conversion factors to calculate a female’s total effective risk for an annual, biennial, or triennial screening program, for a commencement age anywhere from 25 to 75 years, assuming a constant screening cessation age at 75 years. The LER for biennial screening from 40 to 75 years from M.Ali et al. versus our model was 19.38 and 25.61 induced breast cancers per 100,000 females screened, respectively.

It is clear that the breast cancer incidence results from this study are relatively consistent with previous literature, when adapted to fit these previous parameters; however, it is interesting to note that the death rates from this study are consistently a little higher. Not only does this work support existing theories, but it also improves upon them by providing an individualised approach to dose calculation for each age of screening, using only the information from the female’s first screening visit. In addition to that, this experiment provides several new and improved insights into the relationship between females’ breast characteristics and examination exposure parameters. This includes a) the relationship between MBD and CBT with age (Figures 1 and 2), and b) the relationship between the female’s CBT and the various exposure factors used within a Hologic system, such as the kVp, mAs, and T/F combination (Figures 3 and 4, and Table 2).

### Limitations

RRIMS aims to introduce a novel and detailed approach to the dose and risk calculation involved with screening mammography, and as such there are a few limitations. This is mostly a consequence of the inherent unavailability of key pieces of information within the dataset and a frequent lack of associated medical physics QA reports for each available X-ray machine, which are integral to calculate the MGD for each examination. Given the numerous new developments within this paper, all of which aim to achieve an accurate MGD across all ages of screening, the main limitations are best described with the two major filtering criteria that were required when assessing the suitability of the images for their use in this study. First, percentile curves were introduced when assessing the relationship between MBD with age; an improvement over the prototype^8^ from using one average MBD curve along with a correction factor. However, since females’ MBD were not readily available in the DICOM file, it had to be measured using a third-party software (LIBRA), which is only currently trained on Hologic and GE data. This criterion alone meant that the majority of the images within the Lifepool dataset could not be accurately relied upon so only the Hologic images were utilised in this study. Second, a new method was developed to reliably determine what exposure factors would be used for each image; in essence predicting what exposure (kVp and mAs) and T/F combination the mammography machine would give based on the female’s breast characteristics (Step 3c and 3d). When analysing the data, it was evident that CBT is the best predictor of kVp and T/F selection (Figure 3), in addition to being a very good predictor of mAs selection (Figure 4). However, this method would only reveal this CBT and exposure factor relationship with images taken from Hologic, Fujifilm, and Siemens (model: Inspiration) images, and not Philips, Sectra, Konica, GE, and Siemens (model: Novation). As a result, considering the various manufacturers within both the CINSW and Lifepool datasets, the common denominator of manufacturer whose images were compatible with the selective requirements for this study, was Hologic. This is ultimately why we decided to make RRIMS a Hologic focused model for now, with the aim of later expanding into a multivendor approach by adding compatibility for different manufacturers.

We also recognise the limitations with the LAR values that were used to calculate the risk. When attempting to calculate the LER, we could have used the female’s individualised calculated dose in combination with the age-associated LAR values to calculate her individual lifetime risk. However, we avoided this because the LAR is meant for a population level analysis of cancer incidence and mortality. In addition, when calculating the risk of radiation-induced breast cancer from screening mammography, it is important to note that there are other personal factors outside of radiation that would need to be considered such as the female’s ethnicity, body mass index (BMI), hormone replacement therapy (HRT) use, menopause status, parity, and age at first birth.^19^

## Conclusion

This model has demonstrated the ability to provide, in an easily understandable manner, the total screening dose and risk profile of a female, using only the information from her first screening visit. This will further assist females with their informed consent discussion with their medical advisors, with regards to attendance at breast screening programs. It can also inform policy makers when assessing the benefits and drawbacks of various screening patterns and frequencies, along with the appropriate start and end ages recommended for regular screening mammography.

Future iterations of RRIMS will aim to include other manufacturers. The next stage of this study is to validate the outputs to assess the accuracy and consistency of the model. This will be achieved by comparing the doses predicted by our model from a female’s first screening visit with the doses she would actually receive having undergone multiple screening attendances.
